# The Systemic Link Between Oral Health and Cardiovascular Disease: Contemporary Evidence, Mechanisms, and Risk Factor Implications

**DOI:** 10.3390/diseases13110354

**Published:** 2025-10-31

**Authors:** Florinel Cosmin Bida, Florin Razvan Curca, Raoul-Vasile Lupusoru, Dragos Ioan Virvescu, Mihaela Scurtu, Gabriel Rotundu, Oana Maria Butnaru, Teona Tudorici, Ionut Luchian, Dana Gabriela Budala

**Affiliations:** Grigore T. Popa University of Medicine and Pharmacy, 700115 Iasi, Romania

**Keywords:** periodontal diseases, cardiovascular diseases, oral microbiota, systemic inflammation, endothelial dysfunction, atherosclerosis, oral health

## Abstract

Background: Oral health plays a critical role in systemic wellbeing, with growing evidence supporting strong associations between oral conditions and cardiovascular disease (CVD). These connections extend beyond periodontal disease and involve oral microbiota imbalance, systemic inflammation, and oral side effects of cardiovascular pharmacotherapy. Objective: To explore these links, a narrative literature review was performed using PubMed, Scopus, and ScienceDirect, covering studies published between 2000 and 2025. Methods: A comprehensive literature search was conducted in PubMed, Scopus, and ScienceDirect for studies published between January 2000 and May 2025. Both MeSH and free-text terms related to oral health, periodontal disease, systemic inflammation, endothelial dysfunction, and atherosclerosis were used. Eligible studies included observational and interventional research, systematic reviews, and meta-analyses. Key findings: The evidence consistently supports an association between chronic periodontal inflammation and cardiovascular risk, mediated by systemic dissemination of proinflammatory cytokines (IL-6, TNF-α, CRP) and microbial products that promote endothelial activation and atherogenesis. Interventional data indicate that periodontal therapy may reduce systemic inflammatory burden and improve vascular parameters, though heterogeneity across studies limits causal inference. Conclusions: Current findings highlight a significant oral–systemic connection through inflammatory and endothelial mechanisms. Strengthening interdisciplinary collaboration between dental and cardiovascular care providers is essential to translate this evidence into preventive and therapeutic practice. Further longitudinal and mechanistic studies are required to confirm causality and guide clinical integration.

## 1. Introduction

In spite of advances in medical care, cardiovascular disease (CVD) continues to claim the lives of about 18 million people every year, making it the top cause of death globally [1]. Cardiovascular disease accounts for 31% of all fatalities worldwide, as reported by the World Health Organization [1,2]. In addition to a personal or family history of cardiovascular disease, other risk factors include diabetes, high blood pressure, high cholesterol, smoking, insufficient physical activity, obesity, and an unhealthy diet [3]. Periodontal disease is another possible risk factor for CVD, according to increasing data [4,5,6].

Because of its special role in social interactions, including expression of emotion and communication—and in survival—through mastication and the production of protective reflexes that shield other internal homeostatic systems (such as the cardiovascular system and digestive tracts) from harmful environmental changes, the orofacial region is distinct. Consequently, sound oral care in this area is crucial to general health.

At its core, the oral–systemic health link is about a person’s ability to eat, talk, and socialize without discomfort or shame. Each person’s health and capacity to make a positive impact on society are enhanced by these skills. Some systemic disorders might have their roots in poor oral health, and vice versa. This is especially true when it comes to the connections between orofacial disease and conditions including diabetes, heart disease, pneumonia, and rheumatoid arthritis [7]. In addition, the mouth is a model system for other parts of the body, and its intricate biological ecology may teach us a lot about the mechanisms underneath disease [7,8].

Nonetheless, there has been a lot of focus on the systemic effects of dental health on cardiovascular disease in the last several years. As the “gateway to the body,” the mouth is important for more than just eating and talking; it also regulates the body’s overall health. There is growing evidence that inflammation, immunology, and microbiology play a role in the development of cardiovascular illnesses, and that poor dental health may be a major contributor to these processes [8,9].

Tooth loss and self-rated gum issues were found to be indicators of an elevated risk of ischemic heart disease in recent Australian research comprising 172,630 adults with CVD [10]. Another research that included 60,174 adults in the Netherlands clinically assessed periodontitis and discovered that it had an independent link with atherosclerotic CVD [11]—this one employed proxy measures like tooth loss for periodontitis. Systematic reviews and meta-analyses have also found similar relationships [12,13,14,15,16,17].

There is worldwide agreement that patients with CVD should be informed about the significance of oral health and offered a risk assessment and a referral to a dentist [18], even though there is no evidence that periodontal treatment improves cardiovascular outcomes [19,20,21]. Nurses already working in cardiac care have additional opportunities to promote preventive dental healthcare, according to a recent scoping assessment [22].

Global variation in the prevalence and severity of periodontitis—driven by differences in genetics, microbiota composition, diet, and access to oral healthcare—appears to parallel regional disparities in cardiovascular disease burden. Populations with higher rates of periodontal inflammation, such as those in parts of Eastern Europe, South America, and Southeast Asia, also report elevated cardiovascular morbidity and mortality, suggesting that shared lifestyle and inflammatory risk factors contribute to this geographic overlap [23].

Patients’ oral health and overall wellbeing can be adversely affected by a number of cardiovascular medicine side effects, including xerostomia, gingival enlargement, taste disturbances, and mucosal bleeding [22,23,24,25,26]. These effects are common among antihypertensives, anticoagulants, and lipid-lowering agents [27,28].

Evidence suggests that cardiovascular treatment should encompass dental health as well. The lack of oral health knowledge and the difficulty of obtaining dental treatment are two of the major reasons why so few people with CVD actually see the dentist, according to worldwide surveys [29,30].

This review was designed as a structured narrative synthesis, integrating mechanistic, translational, and clinical evidence on oral–systemic interactions. The narrative format was chosen to accommodate heterogeneous data from basic, preclinical, and clinical studies, which could not be analyzed under a systematic or scoping framework without loss of conceptual coherence. Rather than focusing on statistical effect sizes, this narrative review emphasizes the conceptual frameworks and pathophysiological mechanisms that emerged across studies.

Unlike previous reviews that focused separately on epidemiological or microbiological aspects, the present narrative synthesis provides an integrated interpretation of contemporary evidence connecting oral and cardiovascular health. It uniquely combines data from clinical, molecular, and mechanistic studies to outline how oral inflammation and microbial dissemination contribute to endothelial dysfunction and systemic vascular risk. By bridging basic science and clinical outcome, the main objective of this review is to analyze and summarize current knowledge on the relationship between periodontal inflammation, endothelial dysfunction, and cardiovascular outcomes, emphasizing shared biological mechanisms and clinical implications.

## 2. Literature Review

As a major public health concern that impacts many people and places a heavy financial strain on nations, oral illnesses are far from over. Both of these factors are strongly linked to non-communicable disease, which exacerbate health disparities since they disproportionately affect low-income and socially vulnerable groups [31].

In high-income nations, the impact of oral disorders is still a major problem, reducing productivity and quality of life. This is rather concerning. Since a lot of dental problems may be avoided, the World Health Organization has set a lofty target of better oral health worldwide by 2030 [32]. Addressing the rising incidence of non-communicable disease is an integral part of its larger objective to attain universal health coverage, which includes removing financial barriers to quality treatment for everyone [33].

➢ Search Strategy

To synthesize the current evidence on the systemic link between oral health and cardiovascular disease, we categorized the literature into key thematic areas based on recurring patterns and biological relevance. The study was conducted as a narrative review, aiming to synthetize current evidence regarding the systemic relationship between oral health and cardiovascular diseases. Rather than focusing on statistical effect sizes, this narrative review emphasizes the conceptual frameworks and pathophysiological mechanisms that emerged across studies.

The literature search was conducted in PubMed, Scopus, and Web of Science, covering studies published between 2000 and 2024. Search terms combined MeSH descriptors and free-text keywords. Articles were screened by relevance to the topic and methodological quality, with exclusion of non-English and grey literature. Oral health, oral symptoms, cardiovascular disease, periodontal disease, oral microbiota, systemic inflammation, endothelial dysfunction, and atherosclerosis were some of the most common search phrases. To provide the highest level of sensitivity and specificity, the search phrases were customized for each database. This structured narrative approach, combined with explicit inclusion criteria and an informal quality assessment, ensured transparency and reproducibility consistent with current methodological standards for narrative synthesis.

➢ Inclusion and Exclusion Criteria

Inclusion criteria were:

✓ Peer-reviewed articles published in English between January 2000 and May 2025;
✓ Studies involving adult human subjects;
✓ Original research (observational or interventional), systematic reviews, or meta-analyses examining links between oral and cardiovascular health.

Exclusion criteria were:

✓ Case reports, editorials, and conference abstracts;
✓ Studies focusing solely on pediatric populations;
✓ Papers not directly addressing the oral–cardiovascular connection.

➢ Study Selection and Quality Appraisal

Two separate reviewers were responsible for making the final selection. The GRADE (Grading of Recommendations, Assessment, Development, and Evaluation) method was used informally to assess the methodological quality of the clinical trials and systematic reviews. Assessing the methodological quality of the included studies helped to identify the most reliable data and avoid overinterpretation of heterogeneous results. The informal GRADE-based quality assessment provided a structured way to interpret the strength of evidence while maintaining the integrative nature of the review.

➢ Data Synthesis

In this review, studies were interpreted according to their hierarchical weight of evidence. Observational and cross-sectional studies were primarily considered as sources of associative data supporting potential links between oral and cardiovascular conditions, whereas randomized controlled trials and systematic reviews were regarded as higher-level evidence confirming causal or mechanistic relationships. This differentiation allowed for a more balanced and critical interpretation of the available findings.

The reviewed literature reveals several critical gaps that limit comprehensive understanding of the oral–cardiovascular connection. Despite an increasing number of cross-sectional and case–control studies, there is a lack of long-term prospective trials capable of confirming causal associations between periodontal inflammation and cardiovascular outcomes. Many existing studies suffer from heterogeneous diagnostic criteria, inconsistent measurement of inflammatory and endothelial biomarkers, and limited adjustment for key confounders such as age, smoking, and metabolic comorbidities. Furthermore, sex-specific and population-based analyses remain underrepresented, reducing external validity. Only a few studies explore mechanistic pathways through molecular or microbiome profiling, indicating the need for integrative research that combines clinical, biochemical, and genetic perspectives.

Although most available studies support an association between oral and cardiovascular diseases, several methodological limitations and biases must be acknowledged. Considerable heterogeneity exists in study design, with cross-sectional data often overrepresented compared to longitudinal or interventional evidence. Diagnostic criteria for periodontal disease differ across studies, making direct comparisons difficult and potentially introducing classification bias.

Many studies rely on self-reported medical history or incomplete adjustment for confounders such as smoking, diabetes, or socioeconomic status, which may lead to residual confounding. In addition, sample sizes are frequently small and populations geographically limited, restricting generalizability. Publication bias is also possible, since positive associations are more likely to be reported than null findings. Recognizing these issues is essential for accurate interpretation and for guiding future research toward stronger methodological designs.

Each subsection summarizes evidence regarding a specific dimension of the oral–cardiovascular relationship, including inflammatory pathways, microbial translocation, medication-related oral effects, and therapeutic outcomes, as seen in Table 1 below:

### 2.1. Systemic Inflammation (Elevated CRP, IL-6, TNF-α)

The biological pathways underlying the oral–cardiovascular connection are centered on the systemic effects of chronic inflammation and microbial dissemination. The biological pathways connecting oral inflammation and cardiovascular disease involve a sequential interaction between microbial invasion, immune activation, and vascular response. Periodontal pathogens and their endotoxins can reach the bloodstream, initiating systemic inflammation through the release of cytokines such as IL-6, TNF-α, IL-1β, and CRP. These mediators promote endothelial activation, increase oxidative stress, and impair nitric oxide-dependent vasodilation, leading to endothelial dysfunction and progressive atherogenesis. This cascade provides the physiological foundation for the observed clinical association between periodontal and cardiovascular diseases [34,35]. Although periodontal disease has received much of the focus as a possible risk factor for CVD, the scope of oral–systemic interactions is far broader [36].

An increasing amount of evidence is pointing to a connection between periodontal disease and other health problems [38]. The ageing population in industrialized nations is retaining more of their natural teeth, which is leading to an increase in the prevalence of periodontal disease [41]. In addition to increasing the risk of cardiovascular disease and other major noncommunicable diseases, this problem frequently results in tooth loss.

Many pathways have been identified as potential connections between periodontal disease and cardiovascular disease. Plaque activation by oral bacteria such as Streptococcus mutans and Streptococcus sanguinis can lead to thrombogenesis, inflammation, atherogenesis, and the formation of localized thrombus.

**Figure 1:**
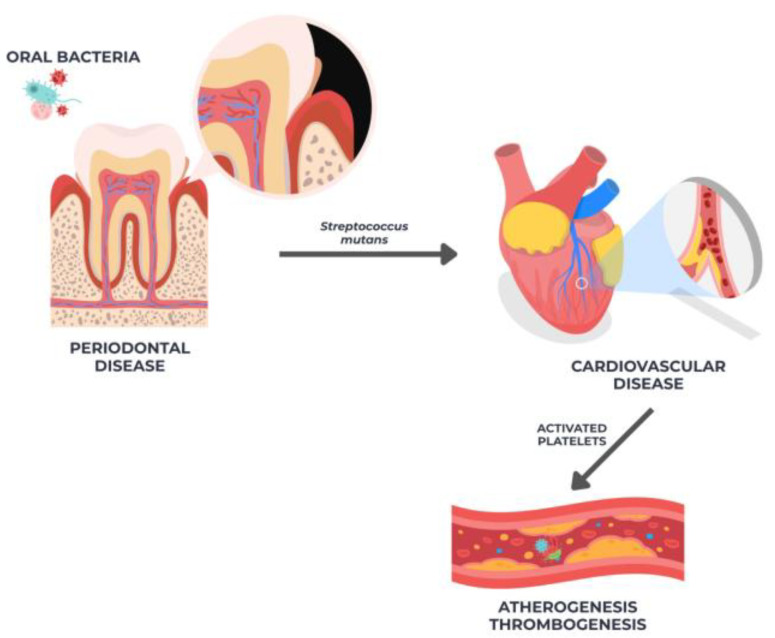
Illustration of the link between periodontal disease and cardiovascular disease through bacterial-induced platelet activation, systemic inflammation, atherogenesis, and thrombogenesis.

Additionally, platelets can emit pro-inflammatory cytokines [43,45]. Systemic inflammation may explain the correlation between periodontitis and cardiovascular disease. Figure 1 below shows the correlation between inflammatory cytokines (IL)-1, IL-6, and TNF-a, acute phase proteins (CRP), and inflammatory cytokines (IL)-1, IL-6, and TNF-a, which are all highly associated with an elevated risk of cardiovascular disease [50,51]. These pro-inflammatory mediators act synergistically to impair vascular homeostasis. Interleukin-6 (IL-6) promotes hepatic synthesis of C-reactive protein (CRP) and fibrinogen, amplifying systemic inflammation and increasing blood viscosity. Tumor necrosis factor-alpha (TNF-α) and interleukin-1β (IL-1β) stimulate endothelial cells to express adhesion molecules such as VCAM-1 and ICAM-1, which recruit circulating monocytes to the vascular wall. Within the intima, these monocytes differentiate into macrophages, ingest oxidized lipoproteins, and form foam cells, initiating fatty-streak development. Meanwhile, CRP directly reduces nitric oxide (NO) bioavailability by inhibiting endothelial nitric oxide synthase (eNOS), causing vasoconstriction and oxidative stress. The resulting cascade of endothelial activation, leukocyte adhesion, and lipid deposition constitutes the biological foundation of atherogenesis in individuals with chronic periodontal inflammation.

Furthermore, it has been proposed that immune cells are primed in a chronically inflamed periodontium, which makes them more likely to be recruited to perivascular tissues through chemotactic mechanisms. This recruitment process eventually leads to the development of hypertension and atherosclerotic disease [44,45].

The mechanistic link between systemic inflammation and cardiovascular dysfunction is largely mediated by endothelial impairment. Periodontal pathogens such as *Porphyromonas gingivalis* and *Aggregatibacter actinomycetemcomitans* can disseminate into the bloodstream, stimulating the release of IL-6, TNF-α, IL-1β, and CRP. These cytokines activate endothelial cells, upregulating adhesion molecules (VCAM-1, ICAM-1), increasing oxidative stress, and reducing nitric oxide bioavailability. The resulting endothelial dysfunction promotes vascular inflammation, lipid infiltration, and early atherogenic changes. Lipopolysaccharides (LPSs) derived from subgingival biofilms further enhance this process through TLR4-mediated signaling, facilitating monocyte adhesion and foam-cell formation. Clinical evidence demonstrates that patients with advanced periodontitis exhibit higher circulating IL-6 and CRP levels correlated with reduced flow-mediated dilation, confirming the inflammatory–vascular link between periodontal infection and cardiovascular disease [79].

In the analyzed literature, both conventional C-reactive protein (CRP) and high-sensitivity C-reactive protein (hs-CRP) were reported as indicators of systemic inflammation. While standard CRP assays detect acute inflammatory states, hs-CRP provides greater sensitivity for subclinical, low-grade inflammation associated with cardiovascular risk. Therefore, hs-CRP findings were interpreted as reflecting chronic systemic inflammatory activity linked to periodontal disease, whereas conventional CRP results were used primarily to document overt inflammatory responses. This distinction was maintained during data synthesis to ensure a precise interpretation of biomarker-based evidence.

From 2003 to 2018, thirty-one prospective and retrospective studies were analyzed by Munoz Aguilera et al. [97] to summarize the prevalence of hypertension in persons with concomitant periodontitis. An adult periodontitis diagnosis was associated with a greater prevalence of hypertension (range: 7% to 77%), according to 25 out of the 30 studies that were examined [97]. Researchers in both animals and humans have identified systemic inflammation as a key component in the onset of atrial remodeling [49,50,51].

Researchers Chen et al. [98] found that even after accounting for prevalent comorbidities, the incidence of atrial fibrillation was still 31% greater in individuals with periodontitis compared to those without periodontal disease. Additional study has shown that atrial fibrillation is linked to higher levels of dental plaque, bleeding when probing, and periodontal inflammation [75,76].

Inflammatory cells infiltrate the atriums of individuals with atrial fibrillation, lending credence to the idea that systemic inflammation may play a role in the onset of arrhythmia [73,74]. Several investigations have shown that atrial fibrillation patients have elevated levels of inflammatory markers, such as C-reactive protein, tumor necrosis factor-a, and plasma IL-6 [22]. CRP promotes endothelial cell death by downregulating nitric oxide [23]. Research has also demonstrated that TNF has a role in the development of atrial fibrillation by increasing the arrhythmogenicity of the pulmonary veins and causing an imbalance in calcium homeostasis [24]. Multiple investigations have shown that periodontitis patients have elevated levels of these systemic indicators [25,26].

Most of the studies exploring CRP, IL-6, and TNF-α in CVD patients with periodontal disease were observational cohort or case–control designs. According to our GRADE evaluation, this evidence is of moderate to high certainty, given the consistency of biomarker elevation across multiple populations.

### 2.2. Oral Microbiota in Atheromatous Tissue

The oral microbiome has recently come to light as a possible contribution to the pathophysiology of atherosclerosis, which is characterized by persistent infections. Oral infections can have systemic consequences via inflammatory signaling and bacteremia in addition to local periodontal damage. The identification of oral microbial components inside vascular lesions is one exciting topic [52,53,54]. Direct occurrence of oral infections in vascular lesions provides additional strong evidence connecting dental and cardiovascular health beyond systemic inflammation [55].

As a result of periodontal disease, oral microbes can penetrate the bloodstream through the compromised oral mucosa. Then, as they travel via circulation, they reach other tissues [53]. Using a range of detection methods including culture, DNA hybridization, and electron microscopy, it was proven that atherosclerotic plaques included oral bacteria. It was initially documented in 1999 that atherosclerotic plaques can harbor germs that cause periodontal infections. The genomic DNA of periodontal bacteria was found in fifty human atherosclerotic plaques in 2000 by Haraszthy et al. [16]. The detection rate reached 72 percent using PCR (polymerase chain reaction) tests for bacterial 16S rDNA [16].

Animal models have also validated the involvement of oral periodontal bacterial infection in the development of atherosclerosis. The reduction in atherosclerotic plaque mediated by the drop in lipid levels in ApoE-/- mice can be blunted by *P. gingivalis* infection, as shown by Kramer et al. [25]. Based on their findings in a mouse model, Chukkapalli et al. [27] concluded that polyfactorial infection can lead to an increase in plaque area and the progression of aortic atherosclerosis after numerous oral infections with *P. gingivalis*, *T. denticola*, *T. forsythia*, and *Fusobacterium nucleatum*.

Pathogens commonly associated with periodontitis—such as *Porphyromonas gingivalis*, *Tannerella forsythia*, and *Aggregatibacter actinomycetemcomitans*-have been detected through PCR and DNA sequencing in carotid and coronary atheroma [26]. These findings imply a direct microbial contribution to the initiation and progression of atherosclerosis. Mechanistically, the bacteria may reach systemic circulation via ulcerated periodontal pockets, interact with endothelial cells, and trigger an inflammatory response conducive to plaque formation [57,59]. The presence of microbial DNA in atheromatous lesions supports the theory of an infectious component in cardiovascular disease pathogenesis, reinforcing the systemic impact of poor oral health [58].

Beyond the detection of whole bacterial cells in vascular tissues, systemic exposure to microbial components derived from periodontal pathogens plays a crucial role in promoting endothelial injury. Periodontal pockets contain millions of bacteria that release virulent factors including lipopolysaccharides (LPSs), short-chain fatty acids (SCFAs) such as butyrate and propionate, and bacterial DNA fragments. These molecules can cross the disrupted gingival epithelium and enter the bloodstream, where they interact with Toll-like receptors (TLR2 and TLR4) on endothelial and immune cells [79]. Their activation induces cytokine production, oxidative stress, and endothelial dysfunction, even in the absence of viable microorganisms. The detection of *Porphyromonas gingivalis* DNA and LPS in atheromatous plaques supports the systemic translocation of bacterial products as a mechanistic link between oral dysbiosis and atherosclerosis [99].

Periodontal pathogens not only circulate bacterial metabolites and cell wall fragments, but they also actively secrete virulence factors, which is another mechanism that increases vascular damage and the imbalance of the host response. In order to let microbes into the bloodstream, species including *P*. *gingivalis*, *T. denticola*, and *F. nucleatum* release exotoxins and proteolytic enzymes that break down proteins in the extracellular matrix and at the junctions between the cells of the epithelium [100].

These chemicals promote persistent dysbiosis once they enter the bloodstream, where they change immune cell signaling and cause an imbalance in the interactions between the host and bacteria. In addition to contributing to the arterial remodeling mechanisms that characterize early atherogenesis, this change maintains low-grade inflammation [101] and promotes endothelial activation.

Evidence for bacterial DNA in atheromatous plaques derives from molecular case–control studies, which we rated as high quality in the GRADE assessment due to reproducible techniques and consistent detection across different cohorts [102].

### 2.3. Xerostomia Associated with Antihypertensives

Xerostomia, the subjective feeling of dry mouth, is a common side effect of antihypertensive drugs. There is some evidence that some types of antihypertensives, such as diuretics, beta-blockers, calcium channel blockers, and centrally acting medicines, might change the content or decrease the flow of saliva [63]. Thiazides and other diuretics cause fluid loss and dehydration; clonidine and methyldopa, on the other hand, may disrupt the autonomic control of the salivary glands [67]. Too little saliva in the mouth raises the danger of cavities, gum disease, oral candidiasis, and irritation of the mucosal lining [64].

It was only in 2004 research by Nederfors et al. that the severity of xerostomia was examined while using the antihypertensive Bendroflumethiazide [69]. Thiazide and furosemide users had higher xerostomia levels, according to their findings [69].

Messerli et al. reported that hypertension patients taking angiotensin-converting enzyme (ACE) inhibitors, calcium channel blockers, β-adrenergic blockers, and diuretics had a considerably higher percentage of patients experiencing hyposalivation [68].

A statistically significant increase in the proportion and level of xerostomia was observed in the hypertension group in research that gathered data on patients treated with ACE inhibitors, calcium channel blockers, β-adrenergic blockers, and diuretics [68]. Another research found that xerostomia was more common in individuals on antihypertensives, although it didn’t specify which medications were involved [28].

Three more research [63,65,66] also looked at the unstimulated whole saliva flow (UWS) rate and showed that patients using antihypertensives had less saliva than the control group, and all three studies reached statistical significance.

Following therapy with β-adrenergic blockers [28,29] or ACE inhibitors [34], three trials demonstrated an increase in UWS that was not statistically significant. When normotensives were given propranolol and phentolamine, one research found that their UWS dropped significantly [34].

The treatment of xerostomia, which occurs in patients on antihypertensive medication, should be part of standard care for cardiovascular patients. This includes making sure the patient is hydrated, practicing good oral hygiene, and, if needed, using stimulants or salivary replacements [65].

Emerging strategies in the management of xerostomia aim to move beyond symptomatic relief and address the underlying dysfunction of salivary glands. Innovative approaches such as stem cell therapy, bioengineered salivary tissues, and gene modulation show potential for restoring physiological salivary flow [101,102].

Most studies addressing xerostomia in cardiovascular patients were cross-sectional surveys, limiting causal inference. In our GRADE appraisal, this body of evidence corresponds to moderate certainty, emphasizing the need for longitudinal trials.

### 2.4. Gingival Overgrowth from Calcium Channel Blockers

Overgrowth of gingiva is one of the known side effects of calcium channel blockers, especially dihydropyridine derivatives like nifedipine and amlodipine. The disorder is marked by an abnormal growth of gingival connective tissue, which can start in the interdental papillae and spread to a large area of the tooth surface [71]. Reasons cited for this effect include a pro-inflammatory local milieu, drug-induced changes in fibroblast activity, and enhanced collagen production [71,72].

A complex interaction between medication effects and local inflammatory conditions is involved in the etiology of gingival overgrowth caused by calcium channel blockers. Increased production of collagen and glycoproteins reduced collagenase activity resulting in impaired extracellular matrix breakdown, and higher proliferation and activity of gingival fibroblasts are some of the proposed causes.

**Figure 2:**
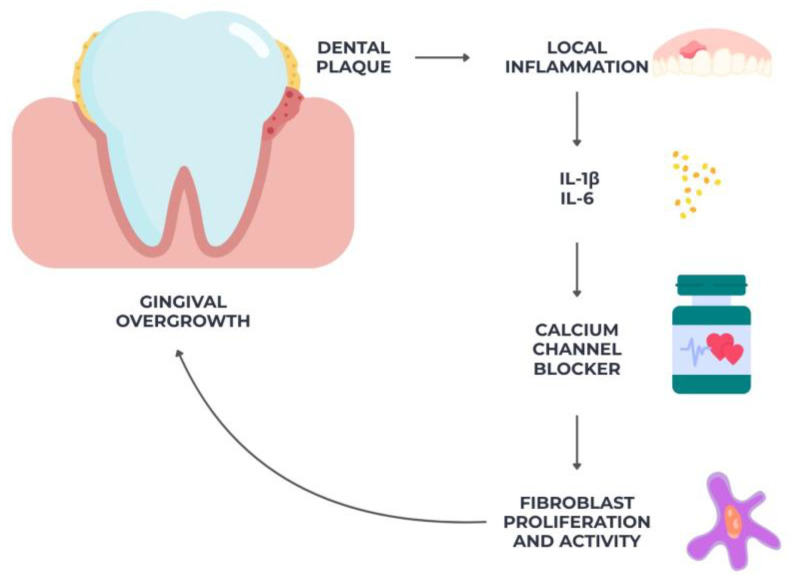
Schematic representation of gingival overgrowth associated with calcium channel blockers, illustrating the interplay between drug-induced fibroblast activation, inflammatory cytokine release, and plaque-induced local inflammation.

Additionally, calcium channel blockers may upregulate profibrotic mediators such as transforming growth factor-beta 1 (TGF-β1), contributing to tissue fibrosis [77]. Local inflammation caused by dental plaque further amplifies this response through the release of pro-inflammatory cytokines such as IL-1β and IL-6, as in Figure 2 below. Altered calcium homeostasis within gingival cells may also influence fibroblast differentiation and function, promoting connective tissue accumulation.

Gingival overgrowth, regardless of its etiology, whether drug-induced or inflammatory—can deepen periodontal pockets and hinder effective plaque control. This altered gingival architecture favors the accumulation of anaerobic, periodontitis-associated bacteria such as *Porphyromonas gingivalis* and *Prevotella intermedia*, thereby amplifying local inflammation and accelerating connective tissue destruction. Consequently, gingival enlargement may act as a secondary risk factor, enhancing microbial dysbiosis and perpetuating the progression of periodontal disease [72,77].

However, the exact process is still not fully understood. An increased risk of complications might result from both hereditary predisposition and neglecting proper dental hygiene [75].

### 2.5. Improved Endothelial Function After Periodontal Therapy

**Figure 3:**
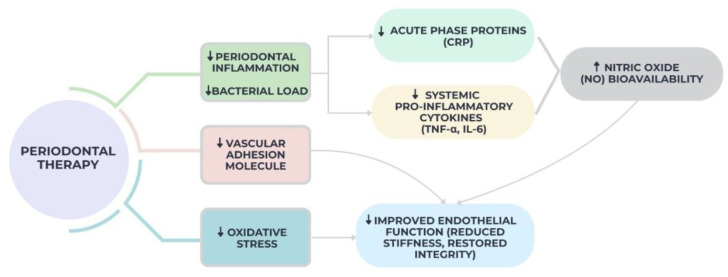
Effect on endothelial function of periodontal therapy.

Emerging evidence suggests that periodontal therapy can improve endothelial function through both local and systemic mechanisms [80]. By reducing periodontal inflammation and bacterial load, treatment diminishes systemic levels of pro-inflammatory cytokines such as IL-6 and TNF-α, as well as acute-phase proteins like CRP [80,81,82]. This systemic anti-inflammatory effect appears to have direct vascular benefits, particularly in restoring nitric oxide (NO) bioavailability, a key mediator of endothelial-dependent vasodilation [82]. Furthermore, periodontal treatment may reduce oxidative stress and vascular adhesion molecule expression, thereby restoring endothelial integrity and reducing vascular stiffness, as seen in Figure 3 below:

Several interventional studies have confirmed these findings. Tonetti et al. conducted a randomized trial on 120 patients with severe periodontitis, reporting that intensive periodontal therapy led to a 30% improvement in brachial artery flow-mediated dilation (FMD) at six months compared to controls [103].

D’Aiuto et al. observed in a cohort of 94 participants that periodontal treatment significantly reduced serum CRP and IL-6 levels within three months, with parallel improvements in endothelial function as assessed by FMD [2]. Similarly, Higashi et al. showed that in hypertensive patients, periodontal treatment improved endothelial-dependent vasodilation, reducing vascular stiffness markers such as pulse wave velocity [104].

Emerging evidence suggests that the beneficial effects of periodontal therapy may extend beyond local inflammation control to a measurable reduction in systemic microbial products. Several interventional studies have reported decreased serum levels of lipopolysaccharides (LPSs) and bacterial metabolites, such as short-chain fatty acids, following successful scaling and root planing or adjunctive antimicrobial treatment [2,102]. These biochemical changes parallel the improvement in endothelial function and reduction in circulating inflammatory markers, supporting the hypothesis that periodontal therapy may lower systemic microbial burden and mitigate cardiovascular risk.

Evidence for improved endothelial function following periodontal therapy is supported by randomized controlled trials, which we rated as moderate to high certainty in GRADE, although heterogeneity in protocols remains a limitation.

### 2.6. Oral Bleeding and Delayed Healing in Anticoagulated Patients

Oral bleeding is a clinically relevant complication frequently observed in patients undergoing long-term anticoagulant therapy, such as vitamin K antagonists (warfarin) or direct oral anticoagulants. Several observational studies and case series have reported increased bleeding tendency following minor oral surgical procedures, including tooth extractions and periodontal interventions, in anticoagulated patients [86,87,88]. While most bleeding events are self-limiting, they may prolong healing time and increase the need for local hemostatic measures.

Use of anticoagulants has also been linked to slower wound healing, which may be because these drugs interfere with fibrin production at the surgical site and change coagulation cascades [89]. In addition, perioperative oral management is already complicated because of the high prevalence of concomitant cardiovascular diseases, which typically require polypharmacy [90]. Thromboembolic events pose a greater risk than bleeding, so current guidelines advise keeping anticoagulation in place during minor oral surgery with sufficient local control measures [91]. Still, these results show how important it is for cardiologists and dentists to work together to reduce risks to both oral and systemic health.

These results highlight the fact that significant problems are uncommon when suitable preventative measures are used, even though anticoagulation increases the frequency of little oral bleeding and may slow healing. As long as effective local treatments such fibrin sponges, tranexamic acid mouth rinses, or sutures are used, current guidelines advocate continuing anticoagulant therapy during most dental operations [89,90]. Therefore, it is critical to integrate dentistry and cardiac treatment in order to reduce the risks of bleeding and thrombosis.

Most data on oral bleeding in anticoagulated patients come from observational cohorts and retrospective analyses. In our GRADE evaluation, the certainty was moderate, underscoring the need for interventional evidence to optimize management.

### 2.7. Glossodynia and Burning Mouth in CVD Patients

A growing number of cardiovascular disease patients are being diagnosed with glossodynia, which commonly presents burning mouth syndrome [92]. Burning mouth syndrome (BMS) is a chronic orofacial pain condition with a highly variable prevalence depending on study design and diagnostic criteria [93]. Population-based studies report a prevalence between 0.7% and 1.7%, whereas clinical samples may reach 5–7% of middle-aged and elderly individuals, particularly postmenopausal women [105].

This disorder, which may have connections to systemic illness and pharmaceutical treatment, is defined by persistent burning, pain, or dysesthesia in the mouth without the presence of obvious mucosal lesions.

Associations between cardiovascular disease, its pharmacological management, and burning oral symptoms have been suggested but remain poorly documented. Case reports describe burning sensations or glossodynia in patients treated with ACE inhibitors, supporting the hypothesis that certain antihypertensive medications may trigger secondary BMS [106].

The relationship between cardiovascular pharmacotherapy and burning mouth symptoms is documented mainly by case reports and cross-sectional studies. According to our GRADE evaluation, this evidence is of low to moderate certainty, highlighting the need for larger controlled studies.

The current body of evidence reveals several consistent patterns, most notably the strong association between chronic periodontal inflammation and systemic endothelial dysfunction, mediated by proinflammatory cytokines such as IL-6, TNF-α, and CRP. Multiple studies confirm that periodontal therapy can reduce circulating inflammatory markers and improve vascular parameters, suggesting a potential causal link [85].

However, contradictions persist, primarily due to differences in study design, diagnostic criteria for periodontal disease, biomarker variability, and limited control of confounding factors such as smoking or diabetes. Some interventional trials report significant systemic benefits, while others show minimal or short-term effects, reflecting heterogeneity in treatment protocols and follow-up duration [107].

While most of the analyzed literature supports a strong association between periodontal inflammation and cardiovascular disease, the level and quality of evidence remain heterogeneous. Observational and cross-sectional studies form the majority of available data, revealing consistent correlations but not proving direct causality. Systematic reviews and interventional trials, although fewer, strengthen this relationship by showing that periodontal therapy can improve endothelial function and reduce systemic inflammatory markers. Furthermore, potential confounding factors such as smoking, diabetes, or socioeconomic status were not uniformly controlled, which may have influenced the reported outcomes. These considerations underscore the need for cautious interpretation and highlight the importance of well-designed longitudinal studies to validate and refine the current evidence [108,109].

Recent European guidelines on cardiovascular prevention emphasize that chronic inflammation and elevated inflammatory biomarkers, including C-reactive protein (CRP) and interleukin-6 (IL-6), act as risk modifiers beyond traditional factors such as blood pressure, lipids, or smoking status. The SCORE2 and SCORE2-Older Persons (SCORE2-OP) algorithms do not include persistently elevated systemic inflammatory markers or chronic inflammatory conditions. These patients can have a higher actual cardiovascular risk than predicted by standard models. This reinforces the relevance of low-grade systemic inflammation of oral origin as a clinically significant component of cardiovascular risk stratification [110,111].

Beyond CRP, proinflammatory cytokines such as interleukin-1 (IL-1) and interleukin-6 (IL-6) play pivotal roles in the initiation and progression of atherosclerosis and myocardial injury. Elevated circulating IL-6 levels have been consistently associated with adverse cardiovascular outcomes, independent of traditional risk factors [112].

Interventional studies have further demonstrated that targeting these inflammatory pathways can reduce recurrent cardiovascular events. The CANTOS trial showed that selective inhibition of IL-1β with canakinumab led to a significant reduction in major adverse cardiovascular events, supporting a causal link between IL-1-mediated inflammation and atherothrombosis [113].

Similarly, genetic and pharmacological studies on IL-6 receptor signaling revealed that attenuation of IL-6 activity correlates with lower vascular risk [114]. These findings emphasize that systemic inflammatory mechanisms—particularly those involving IL-1 and IL-6—are not only biomarkers but also therapeutic targets, aligning with the observed role of periodontal inflammation in modulating cardiovascular outcomes.

Based on this concept, cardiovascular risk assessment should be routinely performed in patients with periodontal disease and other chronic inflammatory conditions, as their real cardiovascular risk may exceed the levels estimated by the SCORE2 and SCORE2-OP algorithms. Consequently, preventive measures and management of modifiable risk factors—such as hypertension, dyslipidemia, smoking, and metabolic disorders—should be implemented more intensively in these individuals [13].

The relationship between oral and systemic health is bidirectional: on the one hand, periodontal or oral inflammatory diseases act as amplifiers of systemic vascular risk; on the other hand, cardiovascular and metabolic conditions can influence the onset, progression, and treatment outcomes of oral diseases. Integrating oral health evaluation into cardiovascular prevention programs, therefore, represents an essential step toward comprehensive and personalized patient care [115].

## 3. Recommendations for Practice and Research

From a clinical perspective, these findings reinforce the importance of integrating oral and systemic health assessments into daily practice. Regular periodontal evaluation and management should be considered an essential component of cardiovascular prevention strategies, particularly in patients with chronic systemic inflammation or metabolic risk factors. Interdisciplinary collaboration between dentists, cardiologists, and primary care professionals is recommended to optimize early detection and coordinated management of oral–systemic conditions.

Future research should focus on prospective longitudinal studies that clarify causal mechanisms linking periodontal inflammation, endothelial dysfunction, and cardiovascular outcomes. Moreover, interventional trials assessing the impact of periodontal therapy on systemic biomarkers and vascular parameters are needed to strengthen the evidence for clinical translation. Advances in molecular profiling, salivary diagnostics, and microbiome analysis may also provide novel tools to identify at-risk populations and monitor systemic effects of oral therapy. Such multidisciplinary research will contribute to a more personalized and preventive model of healthcare.

## 4. Future Perspectives

The present narrative review synthesized evidence from a heterogeneous body of research, including observational studies, clinical trials, and systematic reviews. As outlined in the methodology, most available data are derived from cross-sectional or case–control designs, which demonstrate associations but cannot confirm causality. This limitation highlights the urgent need for well-powered longitudinal cohort studies and randomized interventional trials to determine whether improving oral health—particularly through periodontal therapy—translates into measurable reductions in cardiovascular events.

Future research should also expand on the mechanistic dimension of the oral–cardiovascular axis. Although systemic inflammation and microbial translocation represent the most consistently reported pathways, further studies integrating multi-omics technologies (metagenomics, metabolomics, proteomics) may clarify how oral microbiota interact with vascular tissues and contribute to endothelial dysfunction [16,51,56]. Similarly, exploration of biomarkers, including salivary inflammatory mediators and microbial signatures, could enable early detection of systemic risk and facilitate artificial intelligence (AI)-based risk prediction models.

Available longitudinal and cohort data indicate that the duration and severity of periodontitis correlate with increased systemic inflammatory activity. Patients with longstanding periodontal disease tend to exhibit higher serum concentrations of CRP, IL-6, and TNF-α compared with periodontally healthy individuals. Moreover, improvements in periodontal status over time are associated with reductions in these markers and enhanced endothelial function, as reflected by flow-mediated dilation measurements [85,106,107]. These findings suggest a time-dependent cumulative inflammatory load that may partially explain the graded relationship between periodontal chronicity and cardiovascular disease risk.

The treatment of xerostomia, gingival overgrowth, and burning mouth symptoms—oral side effects of cardiovascular therapy—is another crucial aspect to consider. Medication adherence and quality of life can be negatively impacted by these illnesses, which are frequently unreported. The incidence and consequences of these problems in big cardiovascular cohorts, as well as methods for prevention or treatment, should be the subject of future research.

New insights into the role of oral infections in systemic disease have emerged because of progress in microbiome research. The processes of microbe translocation and immune priming from the mouth to the bloodstream should be better understood with the use of animal models and metagenomic and metabolomic studies. Furthermore, new routes connecting dysbiosis to systemic inflammation and atherosclerosis may be revealed by integrating mouth microbiome data with gut microbiome analysis.

Discovering reliable biomarkers for the early diagnosis of systemic risk is another significant concern. To improve the accuracy of patient risk stratification, artificial intelligence (AI)-based predictive models could combine salivary and serum inflammatory mediators (CRP, IL-6, TNF-α), endothelial function markers, and microbial signatures. The emergence of such instruments would pave the way for cardiologists and dentists to identify systemic diseases earlier and personalize preventive therapies to each individual.

Finally, to address the global burden of both oral and cardiovascular diseases, research should also focus on integrated care models. Embedding dental health assessments into cardiovascular prevention programs, strengthening collaboration between dentists and cardiologists, and incorporating oral health indicators into electronic health records represent promising directions for improving patient-centered care [18,29,30]. In addition, public health initiatives should prioritize equitable access to oral healthcare, particularly in socioeconomically vulnerable groups, as part of a broader strategy to reduce disparities in cardiovascular outcomes.

This review has several limitations inherent to its narrative design. The heterogeneity of the available literature and the absence of a formal quantitative synthesis may introduce interpretation bias. Only studies published in English and indexed in PubMed, Scopus, and ScienceDirect were considered, which may have led to publication and language bias, excluding potentially relevant research published in other languages or in the grey literature. Nevertheless, this approach was chosen to ensure methodological consistency, data reliability, and accessibility of peer-reviewed scientific sources.

## 5. Conclusions

This narrative review highlights the multifaceted relationship between oral health and cardiovascular disease (CVD).

Stronger evidence supports the role of periodontal inflammation and oral microbiota dysbiosis in systemic inflammatory burden, with elevated mediators such as CRP, IL-6, TNF-α, and IL-1β implicated in endothelial dysfunction and atherogenesis. Microbial DNA from oral pathogens has also been detected in atheromatous plaques, further substantiating the infectious–inflammatory link. Conversely, domains such as burning mouth syndrome, xerostomia, or drug-related gingival overgrowth are supported mainly by case reports and cross-sectional studies, reflecting a lower certainty of evidence.

The transmission of bacterial DNA isn’t the only mechanism that connects periodontal infection to circulatory dysfunction; microbial compounds including lipopolysaccharides (LPSs) and short-chain fatty acids (SCFAs) also play a role. Oral microbial load is a modifiable systemic risk factor because these compounds can circulate at subclinical levels, contributing to chronic endothelial activation, systemic inflammation, and metabolic imbalance.

Clinically substantial issues arise beyond these molecular pathways from oral side effects of cardiovascular treatments. These include xerostomia, gingival overgrowth, oral bleeding, and burning mouth feelings. In addition to lowering quality of life in relation to dental health, these issues may make it harder for patients to stick to their cardiovascular treatment plans.

The originality of this review lies in its multidimensional integration of molecular, clinical, and epidemiological evidence, offering a coherent interpretation of how oral health influences systemic vascular outcomes. This interdisciplinary perspective provides a conceptual foundation for future translational research and preventive strategies.

Furthermore, future research should be well-designed and randomized to validate a causal relationship and determine if better dental health can successfully lower cardiovascular risk. This is because the current studies have limitations.

## Figures and Tables

**Table 1:** Summary of Evidence on the Oral–Cardiovascular Link (GRADE Assessment).

Outcome	No. of Studies	Study Design	Risk of Bias	Inconsistency	Indirectness	Imprecision	Overall Quality
Systemic inflammation (elevated CRP, IL-6, TNF-α) [34,35,36,37,38,39,40,41,42,43,44,45,46,47,48,49,50,51]	18	RCTs+ Cohort Studies	Low	Moderate	No	Moderate	Moderate
Oral microbiota In atheromatous tissue [52,53,54,55,56,57,58,59,60,61,62]	11	Case–Control Studies	Low	Low	No	Low	High
Xerostomia associated with antihypertensives [28,63,64,65,66,67,68,69,70]	9	Cross-sectional Studies	Moderate	Moderate	No	Low	Moderate
Gingival overgrowth from calcium channel blockers [71,72,73,74,75,76,77,78]	8	Observational studies	Moderate	Low	No	Moderate	High
Improved endothelial function after periodontal therapy [79,80,81,82,83,84,85]	7	Clinical Trials	Low	Low	Yes	Moderate	Moderate
Oral bleeding and delayed healing in anticoagulated patients [86,87,88,89,90,91]	6	Observational + case reports	Moderate	Moderate	Yes	Moderate	Moderate
Glossodynia and burning mouth in CVD patients [92,93,94,95,96]	5	Cross-sectional Studies	Moderate	High	Yes	High	Moderate

## Data Availability

Not applicable.

## Abbreviations

The following abbreviations are used in this manuscript:

CVD	Cardiovascular Disease
GRADE	Grading of Recommendations, Assessment, Development, and Evaluation
CRP	C-reactive protein
IL-6	Interleukin-6
TNF-α	Tumor Necrosis Factor-alpha
(IL)-1	Interleukin-1
PCR	Polymerase Chain Reaction
ACE	Angiotensin-Converting Enzyme
UWS	Unstimulated Whole Saliva
TGF-β1	Growth Factor-Beta 1
IL-1β	Interleukin-1 Beta
NO	nitric oxide
FMD	Flow-Mediated Dilation
BMS	Burning Mouth Syndrome
AI	Artificial Intelligence
VCAM-1	Cell Adhesion Molecule-1
ICAM-1	Intercellular Adhesion Molecule-1
SCFAs	Short-Chain Fatty Acids
Toll-like receptors	TLR2 and TLR4
